# Decoding VExUS: a practical guide for excelling in point-of-care ultrasound assessment of venous congestion

**DOI:** 10.1186/s13089-024-00396-z

**Published:** 2024-11-19

**Authors:** Taweevat Assavapokee, Philippe Rola, Nicha Assavapokee, Abhilash Koratala

**Affiliations:** 1 Faculty of Medicine Ramathibodi Hospital, Mahidol University, 270 Rama VI Rd, Thung Phaya Thai, Ratchathewi, Bangkok, 10400 Thailand; 2 Division of Intensive Care, Santa Cabrini Hospital, Montreal, QC Canada; 3 Division of Gynecologic Oncology, Department of Obstetrics and Gynecology, Faculty of Medicine, Chulalongkorn University, 1873, Rama IV Road, Pathum Wan, Bangkok, 10330 Thailand; 4 Division of Nephrology, Department of Medicine, Medical College of Wisconsin, Milwaukee, WI 53226 USA

**Keywords:** Venous excess ultrasound, VExUS, Venous congestion, Point-of-care ultrasound

## Abstract

Venous congestion, often associated with elevated right atrial pressure presents a clinical challenge due to its varied manifestations and potential organ damage. Recognizing the manifestations of venous congestion through bedside physical examination or laboratory tests can be challenging. Point-of-care ultrasound (POCUS) is emerging as a valuable bedside tool for assessing venous congestion, with the Venous Excess Ultrasound (VExUS) technique gaining prominence. VExUS facilitates non-invasive quantification of venous congestion, relying on measurements of the inferior vena cava (IVC) size and Doppler assessments of the hepatic vein (HV), portal vein (PV), and intrarenal vein, thereby providing real-time insights into hemodynamic status and guiding therapeutic interventions. The grading system outlined in VExUS aids in stratifying the severity of congestion. However, achieving proficiency in VExUS requires a comprehensive understanding of Doppler techniques and their clinical applications. This review article provides practical guidance on performing VExUS, encompassing equipment requirements, preparation, machine settings, and examination techniques for assessing the inferior vena cava (IVC), hepatic vein (HV), portal vein (PV), and intrarenal vein. Potential pitfalls and troubleshooting strategies are discussed to ensure accurate interpretation of Doppler waveforms.

**Supplementary Information:**

The online version contains supplementary material available at 10.1186/s13089-024-00396-z.

## Background

Elevated right atrial pressure (RAP) is a major hemodynamic variable in the development of systemic venous congestion [1–3]. When the cardiac pump is overwhelmed by fluid or pressure overload, the venous system bears the burden of increased RAP, resulting in dilation and flow changes detectable through Doppler ultrasound [4–7]. Elevated RAP also impedes lymphatic drainage, further exacerbating tissue congestion. By increasing organ afterload, this sequence of events can trigger detrimental effects, including hemodynamic acute kidney injury (AKI), congestive hepatopathy, intestinal congestion, interstitial edema, and delirium [8–15]. The physical examination frequently lacks reliability in assessing the degree of congestion [16–18]. The incorporation of point-of-care ultrasound (POCUS) for assessing hemodynamics can be advantageous in this scenario and is becoming an essential bedside tool in modern medical practice [19].

Although often simplistically represented as a reflection of elevated RAP, venous congestion results from a complex interplay of venous compliance, circulating blood volume, venous return driven by mean systemic filling pressure (Pmsf), and the compliance and function of the right atrium and right ventricle [1–3]. As such, a comprehensive evaluation of the hemodynamic circuit is imperative for diagnosing congestion and implementing effective management, extending beyond reliance solely on RAP values. The Venous Excess Ultrasound (VExUS) is a POCUS technique used to quantify venous congestion. It begins with measuring the inferior vena cava (IVC) diameter, based on the assumption that the IVC dilates in the presence of elevated RAP. This elevated pressure is subsequently transmitted to systemic veins, leading to altered flow. In the presence of a plethoric IVC, Doppler ultrasound evaluation of the splanchnic (hepatic and portal veins) and renal circulation (intrarenal vein) is performed [20]. Elevated VExUS scores have been linked to organ dysfunction, likely due to decreased perfusion pressure in the context of elevated RAP. Perfusion pressure, grossly defined as the difference between mean arterial pressure and central venous pressure, has been shown to be associated with an increased risk of organ injury when reduced [21]. Furthermore, VExUS not only enables the non-invasive quantification of venous congestion but also allows for real-time assessment of changes with therapy [22–28].

Despite the increasing interest in this sonographic application across various specialties, the current training in POCUS and the assessment of competency display heterogeneity. Many novice POCUS users lack proficiency in both the principles and practical aspects of Doppler techniques. Consequently, errors may occur, ranging from assessing the IVC size to obtaining optimal Doppler images and accurately interpreting them.

In this article, our intention is to offer guidance and methods for executing VExUS accurately while minimizing errors. Relevant images are incorporated as visual aids wherever appropriate.

## Indications

VExUS proves valuable in individuals with suspected volume overload, heart failure, hemodynamic AKI, shock states, or unexplained hypotension, spanning various care environments such as the intensive care unit (ICU), inpatient floors, and ambulatory care settings [22–28]. It is crucial to emphasize that while VExUS offers valuable insights into hemodynamic status, it should not be a substitute for careful history taking and traditional physical examination techniques such as the assessment of capillary refill time or neck veins examination [29]. Instead, it should be integrated into the clinical context to enhance patient care and inform treatment decisions.

## Equipment

VExUS requires an ultrasound system with a two-dimensional image display and Doppler capabilities, specifically color and pulsed wave Doppler. Either a phased array or a curvilinear transducer is suitable for this purpose, though depending on the patient’s body habitus, the use of both may be necessary. For instance, in our experience, the curvilinear transducer is more effective for picking up flows when performing renal Doppler in critically ill patients [30]. It is recommended to have an electrocardiogram (ECG) module connected to the machine for simultaneous ECG tracing with the Doppler waveform to facilitate accurate interpretation [31]. While handheld ultrasound devices can be utilized, there is variability in their image quality, and it is advisable to opt for a high-end device where resources permit. Furthermore, handheld ultrasound devices typically lack ECG capability. Ultrasound transmission gel and gloves should be readily available as with any ultrasound examination.

## Preparation

Before initiating the VExUS examination, universal safety precautions should be adhered to, and appropriate protective gear should be worn in accordance with institutional guidelines, taking into account the patient’s location and infectious concerns. The patient should be positioned in a supine position, and the examination table should be adjusted to the operator’s waist level to ensure optimal scanning conditions. Throughout the procedure, the probe should be handled with one hand, while the ultrasound machine should be operated with the other.

## Machine settings

When evaluating the IVC, hepatic vein (HV), and portal vein (PV), it is appropriate to use standard cardiac, abdominal, or FAST (Focused Assessment with Sonography in Trauma) presets for scanning. While the cardiac preset may provide clearer visualization of these vessels, the color flow for vessel identification may be less distinct compared to the abdominal and FAST presets. Additionally, the cardiac preset may default to higher Doppler scale numbers, such as 100 cm/s; it is essential to reduce it to approximately 40 cm/s and make further adjustments as needed during image acquisition. Some machines also permit ECG tracing only in the cardiac preset, whether using a phased array or the curvilinear transducer. For intrarenal vein Doppler (IRVD) assessment, it is advisable to select the abdominal or FAST preset and set the Nyquist limit (Doppler scale) below 20 cm/s. Adjustments, such as increasing color gain, may be necessary to optimize the visualization of the renal vessels. The Doppler sweep speed for VExUS should be set to 50 or 66.7 mm/s for optimal waveform evaluation. If the Doppler scale is configured too high for a particular vascular area, the absence of color flow detection may lead to an incorrect assumption of reduced flow or thrombosis. On the other hand, setting the scale too low can cause aliasing, which appears as mixed colors on the color Doppler, misleadingly indicating high-velocity turbulent flow that mimics stenosis [30].

It is important to highlight here that, like with any Doppler POCUS application, clinicians performing VExUS should be well-versed in Doppler settings such as wall filter, sample volume size, sweep speed, effects of angle correction, and the measurement tools available on their machines to ensure optimal image acquisition and troubleshooting when needed [32, 33].

## Examination of the IVC

### Technique

**Fig. 1:**
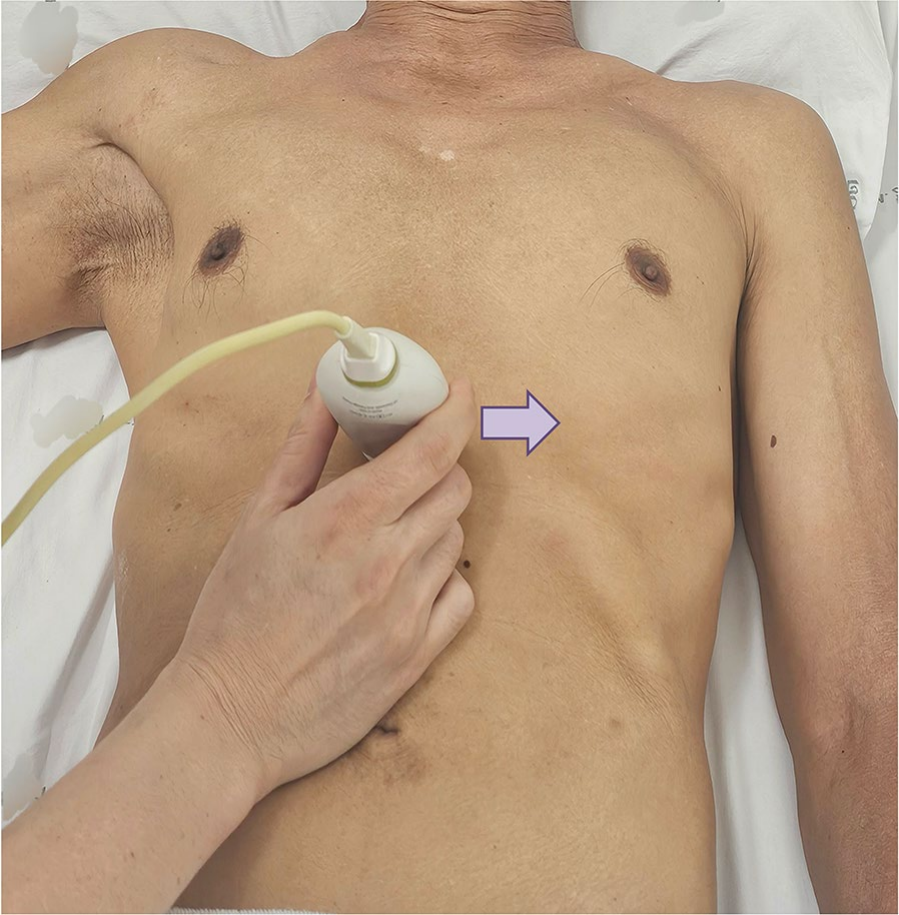
Hand and probe position for the IVC short axis view with the transducer orientation marker set at 3 o’clock (arrow). IVC, inferior vena cava

**Fig. 2:**
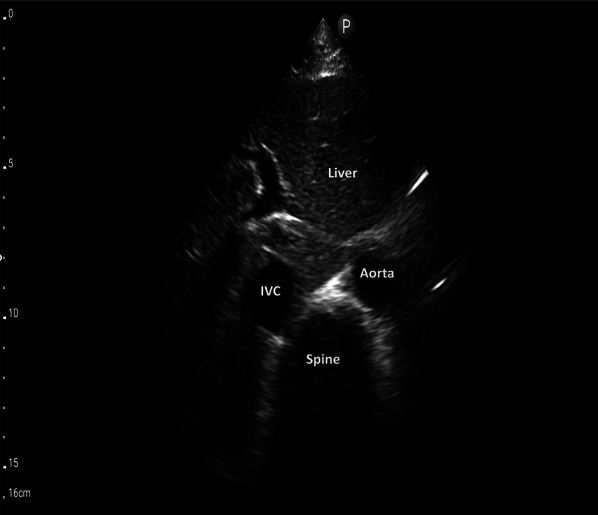
IVC short-axis view. IVC, inferior vena cava

**Fig. 3:**
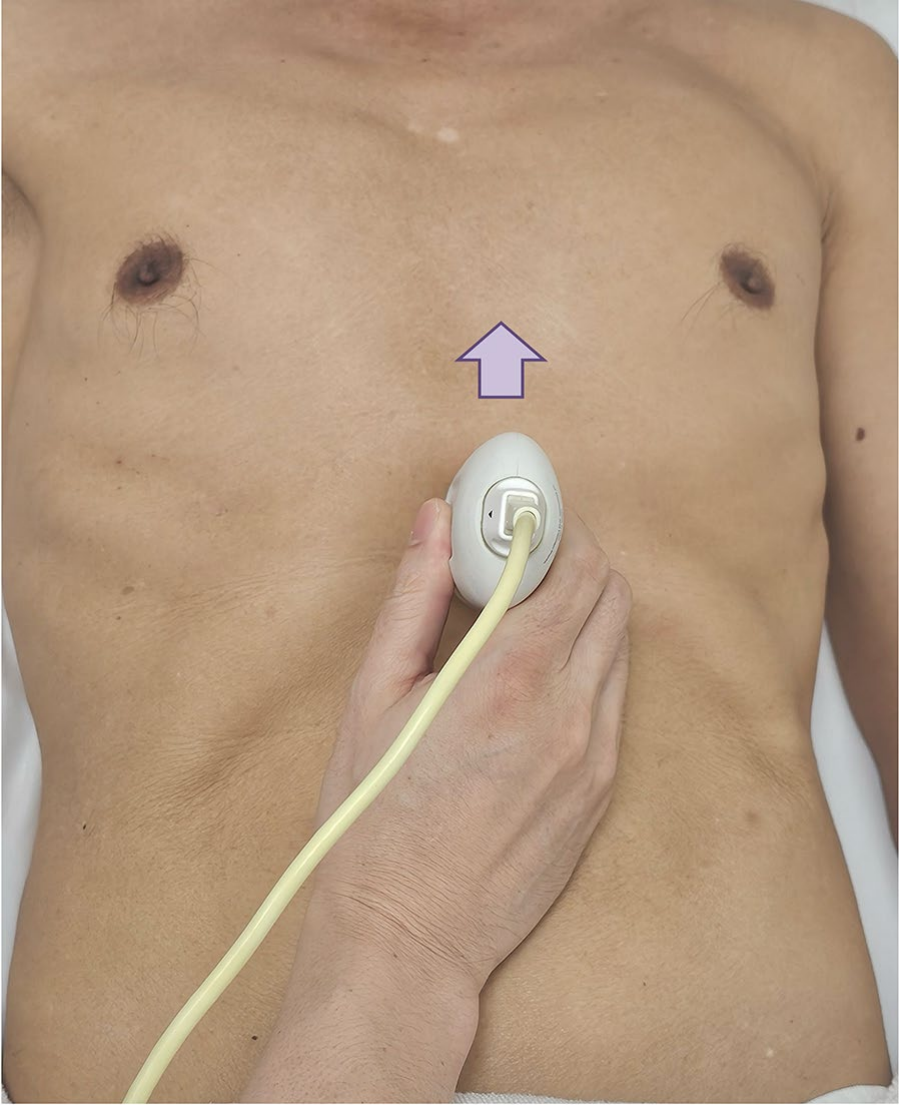
Hand and probe position for the IVC long axis view with the transducer orientation marker set at 12 o’clock (arrow). IVC, inferior vena cava

**Fig. 4:**
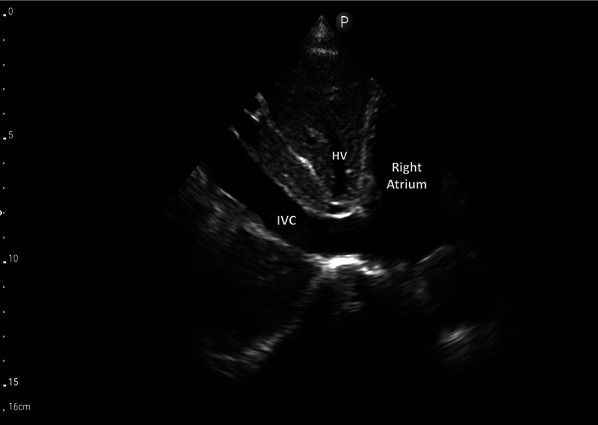
IVC long-axis view. IVC, inferior vena cava; HV, hepatic vein

**Fig. 5:**
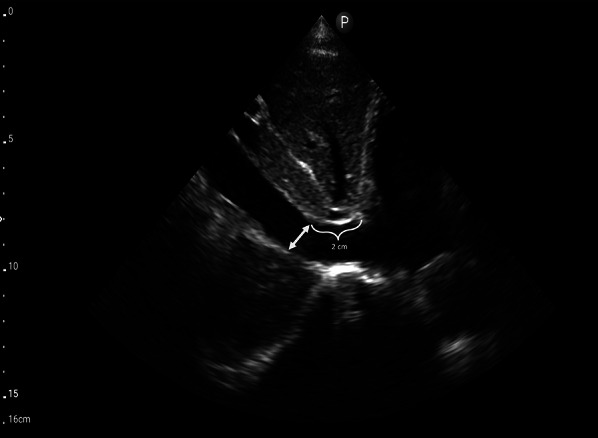
The optimal mesaurement location of the IVC in the long-axis view is either 2 cm below the RA-IVC junction or approximately 1 cm below the HV-IVC junction. IVC, inferior vena cava; RA, right atrium; HV, hepatic vein

**Fig. 6:**
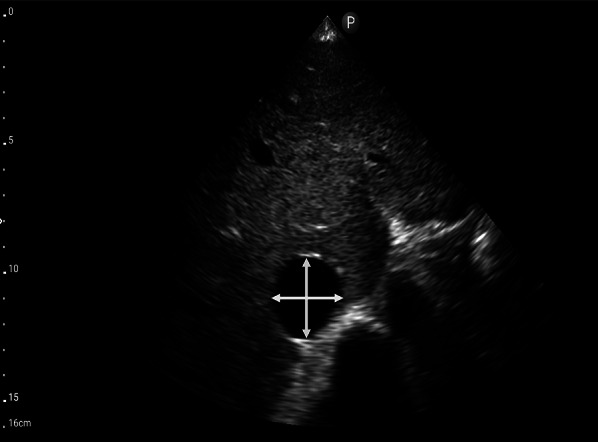
Measurement of the IVC in the short-axis view. IVC, inferior vena cava

The first step of VExUS is to evaluate the maximal diameter of the IVC. Place the transducer 1–2 cm below the xiphoid process with the transducer orientation marker set at 3 o’clock (Fig. 1), capturing the IVC in its short axis through the liver as an acoustic window (Fig. 2; (Video 1). Adjust the transducer closer to the xiphoid process in case bowel gas impedes the view. The abdominal aorta is positioned in the midline separated from the liver, while the IVC is situated to the right within the liver tissue (Fig. 2). Transition to the long-axis view by angling the transducer towards the patient’s right to center the IVC, then rotate the transducer counterclockwise 90 degrees from 3 to 12 o’clock (Fig. 3; Video 2). This rotation enables the visualization of the long axis of the IVC joining the right atrium and the HV draining into the IVC (Fig. 4; Video 3). Some users prefer to image the long axis view first, which is acceptable. When measuring the IVC diameter, choose a location either 2 cm below the right atrium-IVC junction or approximately 1 cm below the HV-IVC junction (Fig. 5) [34, 35]. Refrain from employing M-mode for measurement, as it poses challenges in maintaining control over the M-mode cursor’s position throughout the respiratory phase [36]. Instead, utilize B-mode and freeze the video to obtain precise measurement of the IVC. Some ultrasound machines do offer M-mode that takes respiratory movements into consideration and changes its position accordingly. Alternatively, some experts recommend measuring the IVC in the short axis as well (Fig. 6). Although this differs from the measurement technique used in the original VExUS studies, it is considered a more physiologically sound approach and less susceptible to errors [37]. In an interesting study, CVP showed moderate correlation with short-axis diameter (*r* = 0.69, *P* < 0.001), strong correlation with the short-long ratio (*r* = 0.75, *P* < 0.001), and modest correlation with area (*r* = 0.47, *P* < 0.001), but not with long-axis diameter (*r* = 0.24, *P* = 0.17) [38].

### Interpretation

In VExUS, an IVC maximal size of less than or equal to 2 cm corresponds to VExUS grade 0, indicating the absence of systemic congestion [20]. However, caution should be exercised when applying this threshold to populations beyond the conventional Western demographic, as adjustments to the 2 cm threshold may be necessary. Identifying an IVC that appears plethoric and exhibits a more or less circular shape in the short axis may be more appropriate [38].

### Potential pitfalls

- IVC may be confused with the adjacent aorta. Identify abdominal aorta by observing the aorta’s leftward position, its separation from the liver (Fig. 2), and the presence of anterior branches outside of the liver (such as the celiac and superior mesenteric arteries). It’s important to note that pulsatility should not be solely relied upon for distinguishing these vessels, as the IVC can be pulsatile in hyperdynamic states and tricuspid regurgitation, sometimes more so than the aorta.
- A dilated IVC might be present in endurance athletes without an elevated RAP [39].
- Elevated intra-abdominal pressure can lead to a collapsed IVC despite an elevated RAP [40, 41].
- Solely visualizing the IVC in the long axis can result in the cylinder effect, where the two-dimensional ultrasound beam may cut across the three-dimensional vessel at its periphery instead of the center, leading to inaccurate diameter measurements [34].
- Exclusively visualizing the IVC in the short axis may lead to errors, such as potentially confusing it with the adjacent right atrium or being unable to accurately gauge from the right atrium and locate the appropriate measurement site.

## Hepatic vein Doppler

### Technique

**Fig. 7:**
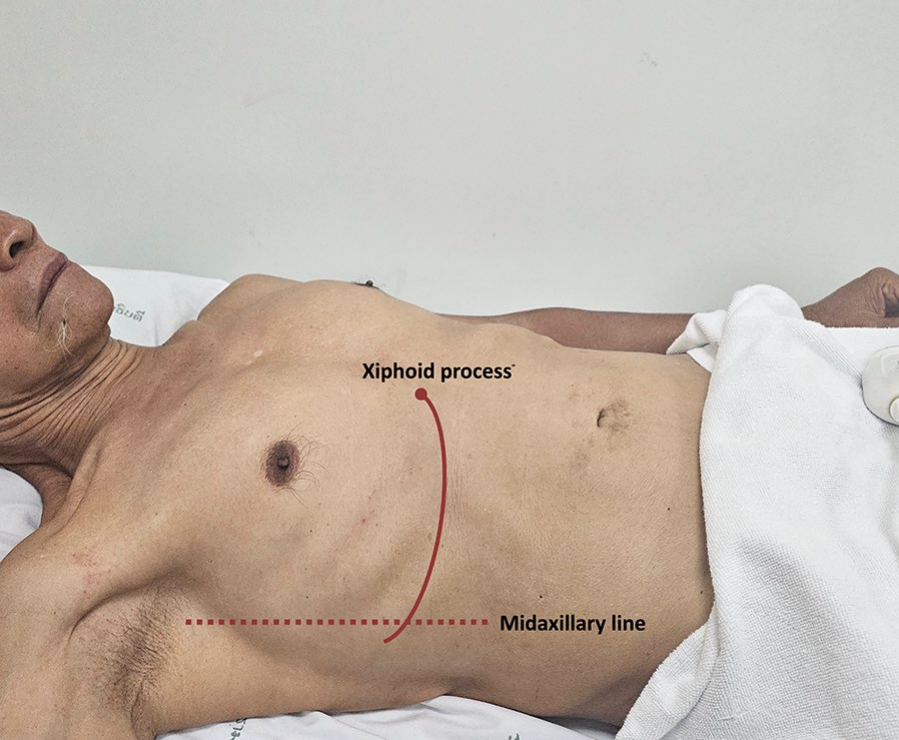
The junction of an imaginary line extending from the xiphoid process to the midaxillary line to visualize the HV in coronal view. HV, hepatic vein

**Fig. 8:**
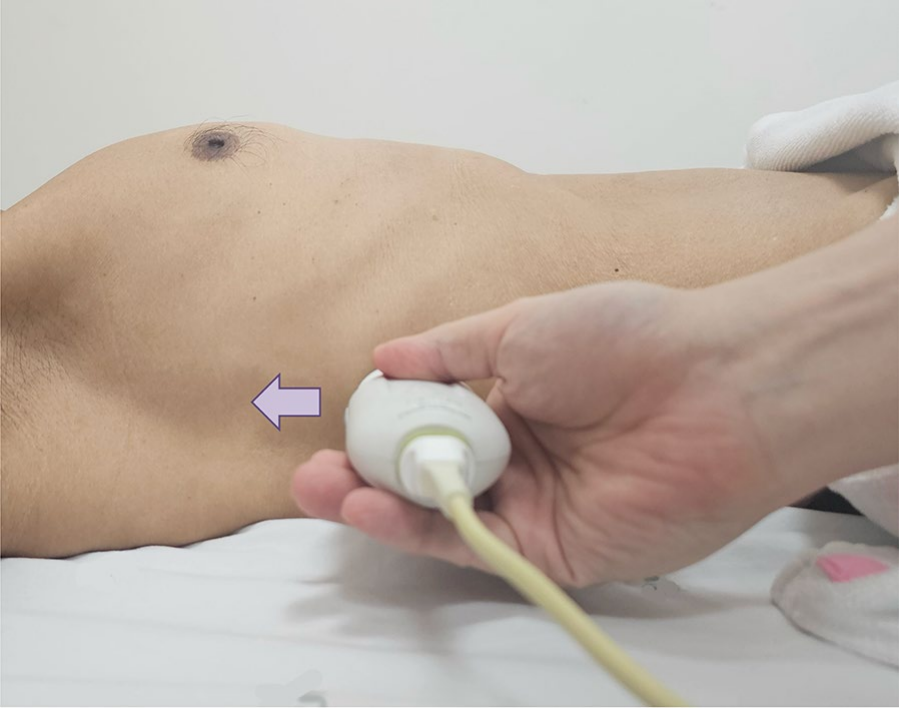
Position the transducer at the junction of an imaginary line extending from the xiphoid process to the midaxillary line, orienting the orientation marker towards the patient’s right axilla (arrow) to visualize the HV in coronal view. HV, hepatic vein

**Fig. 9:**
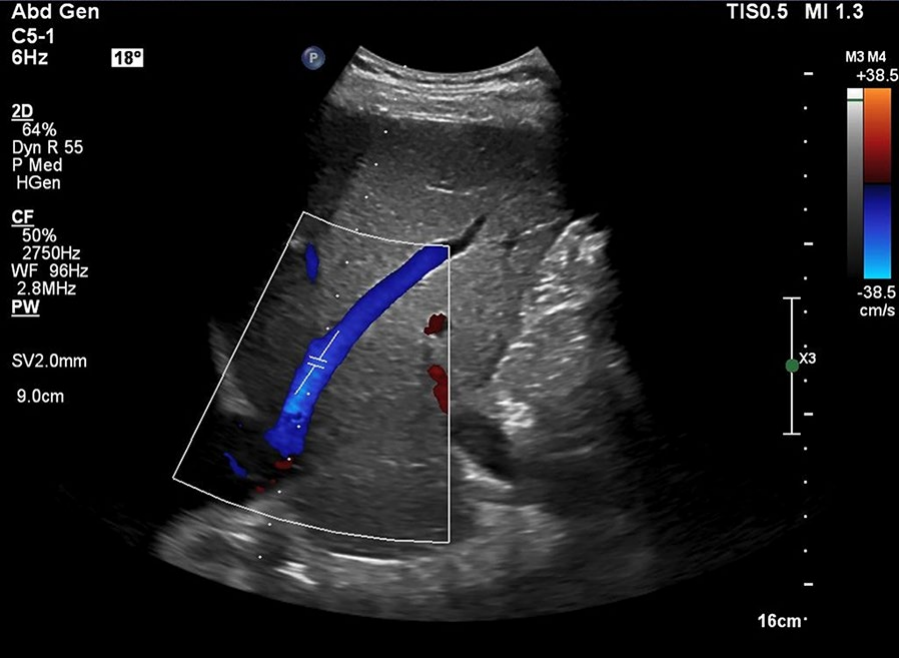
Pulsed wave Doppler mode with the sample volume (Doppler gate) positioned within the HV, approximately 1–2 cm away from its junction with the IVC. IVC, inferior vena cava; HV, hepatic vein

**Fig. 10:**
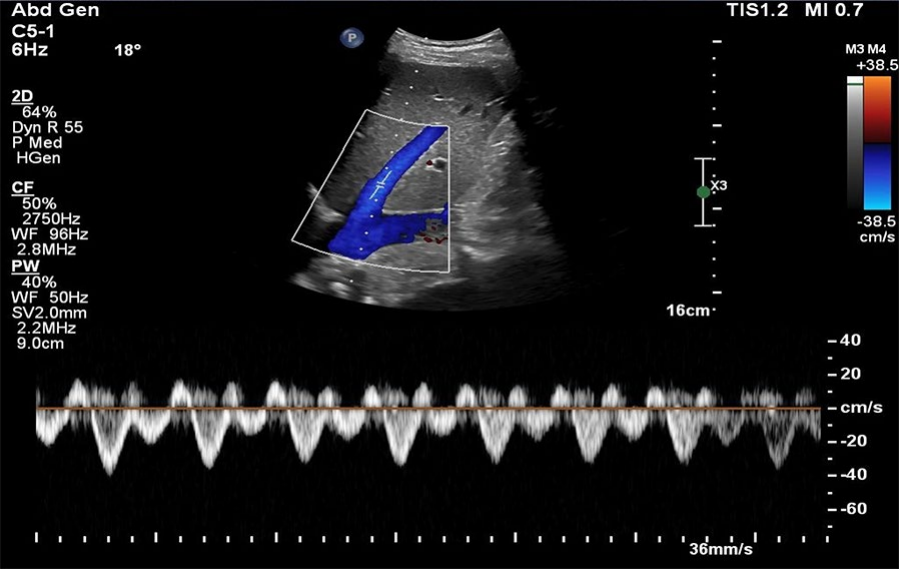
Pulsed-wave Doppler tracing of the HV in abdominal preset. HV, hepatic vein

**Fig. 11:**
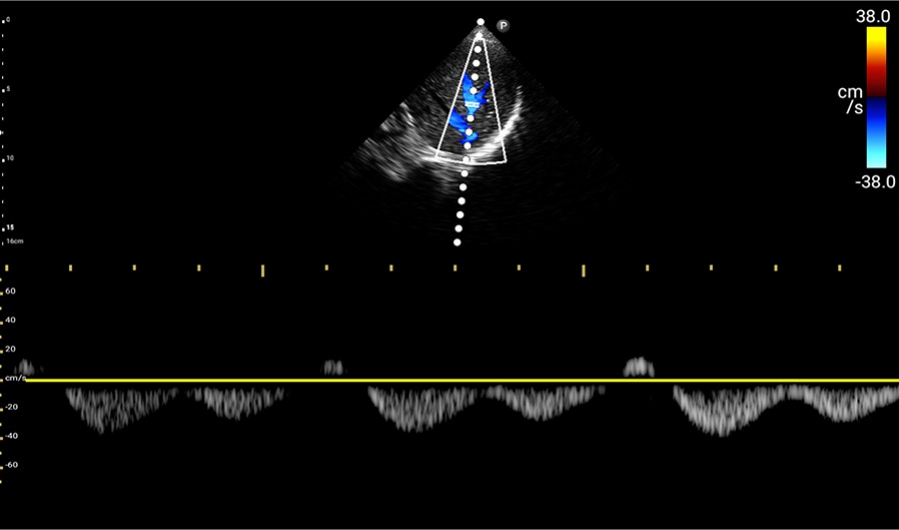
Pulsed-wave Doppler tracing of the HV in cardiac preset. HV, hepatic vein

- Subxiphoid view:
  - Place the transducer 1–2 cm below the xiphoid process with the probe marker oriented towards 12 o’clock (Fig. 3).
  - With a gentle tilt towards the patient’s right, capture the long-axis view of the hepatic vein draining into the IVC (Videos 4, 5).
- Coronal view:
  - This view often results in an optimal waveform compared to that of subxiphoid view.
  - Position the transducer at the junction of an imaginary line extending from the xiphoid process to the midaxillary line (Fig. 7), aligning the orientation marker towards the patient’s right axilla (Fig. 8).
  - Slide the probe slightly towards the patient’s head to visualize the liver- diaphragm interface, then tilt the probe downward to visualize the hepatic vein (Videos 6, 7, 8).
- Doppler assessment:
  - If encountering challenges in visualizing the hepatic vein, the use of color Doppler imaging can assist in locating it (Videos 9, 10).
  - In a normal state, the presence of blue color flow signifies blood moving from the hepatic vein away from the transducer and into the IVC.
  - Engage the pulsed wave Doppler mode, placing the sample volume (Doppler gate) within the hepatic vein at least 1–2 cm away from the junction of the hepatic vein and IVC while being cautious to avoid venous junctions (Fig. 9). Any of the three hepatic veins (right, middle, left) can be sampled based on ease of access.
  - Ability of the patient to hold breath at end-expiration helps. Holding breath at the end of deep inspiration or performing a Valsalva maneuver blunt the waveform.
  - Examine the waveform of the hepatic vein (Figs. 10, 11).

### Interpretation

**Fig. 12:**
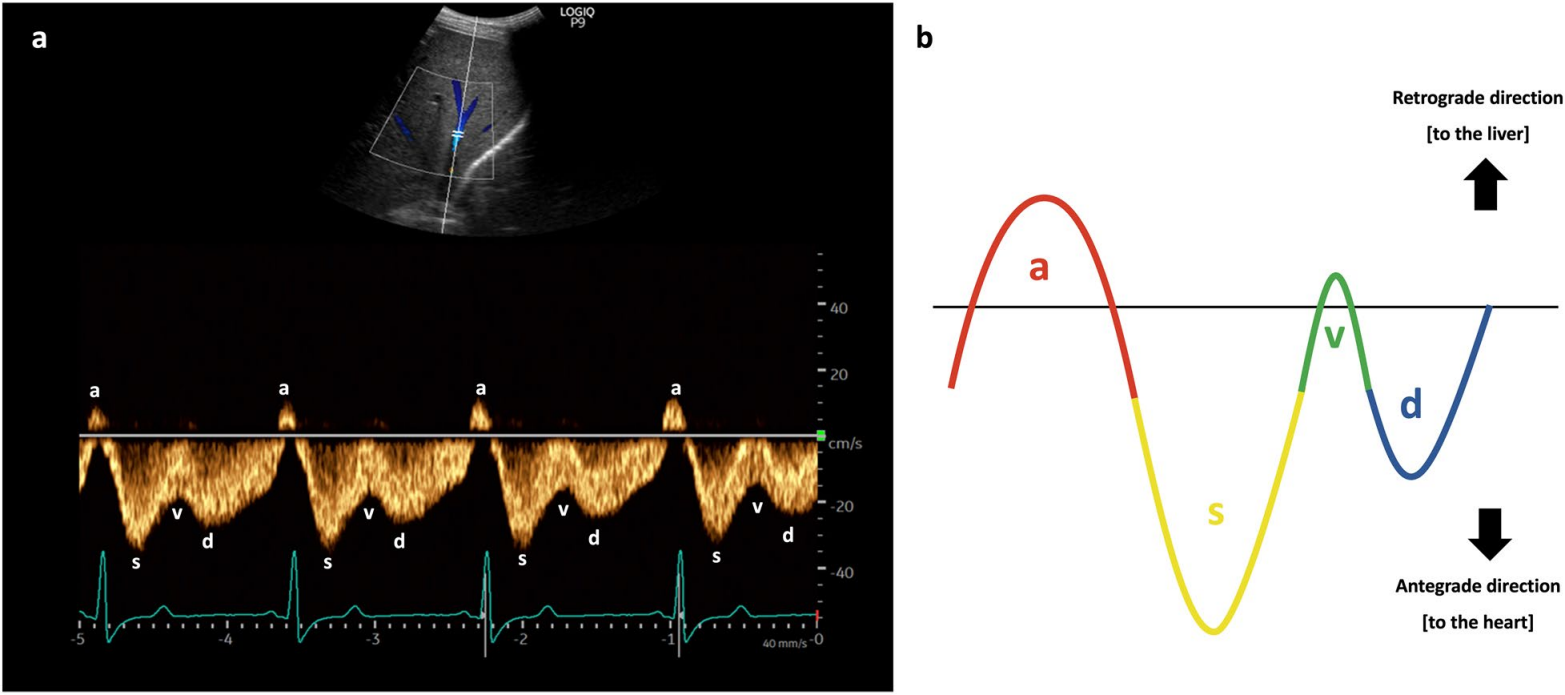
**a** The waveform of the HV, alongside simultaneous ECG, aids in identifying each wave. **b** The waveform of the HV. HV, hepatic vein; ECG, electrocardiogram

The waveform of the hepatic vein comprises two positive retrograde waves (A and V waves) and two negative antegrade waves (S and D waves), indicating blood movement away and towards the heart, respectively [42, 43]. Having a simultaneous ECG assists in identifying each wave: the A wave corresponds to the P wave of the ECG, the S wave aligns with the QRS complex, the V wave occurs at end-systole, and the D wave takes place after the T wave (Fig. 12) [31, 43].

**Fig. 13:**
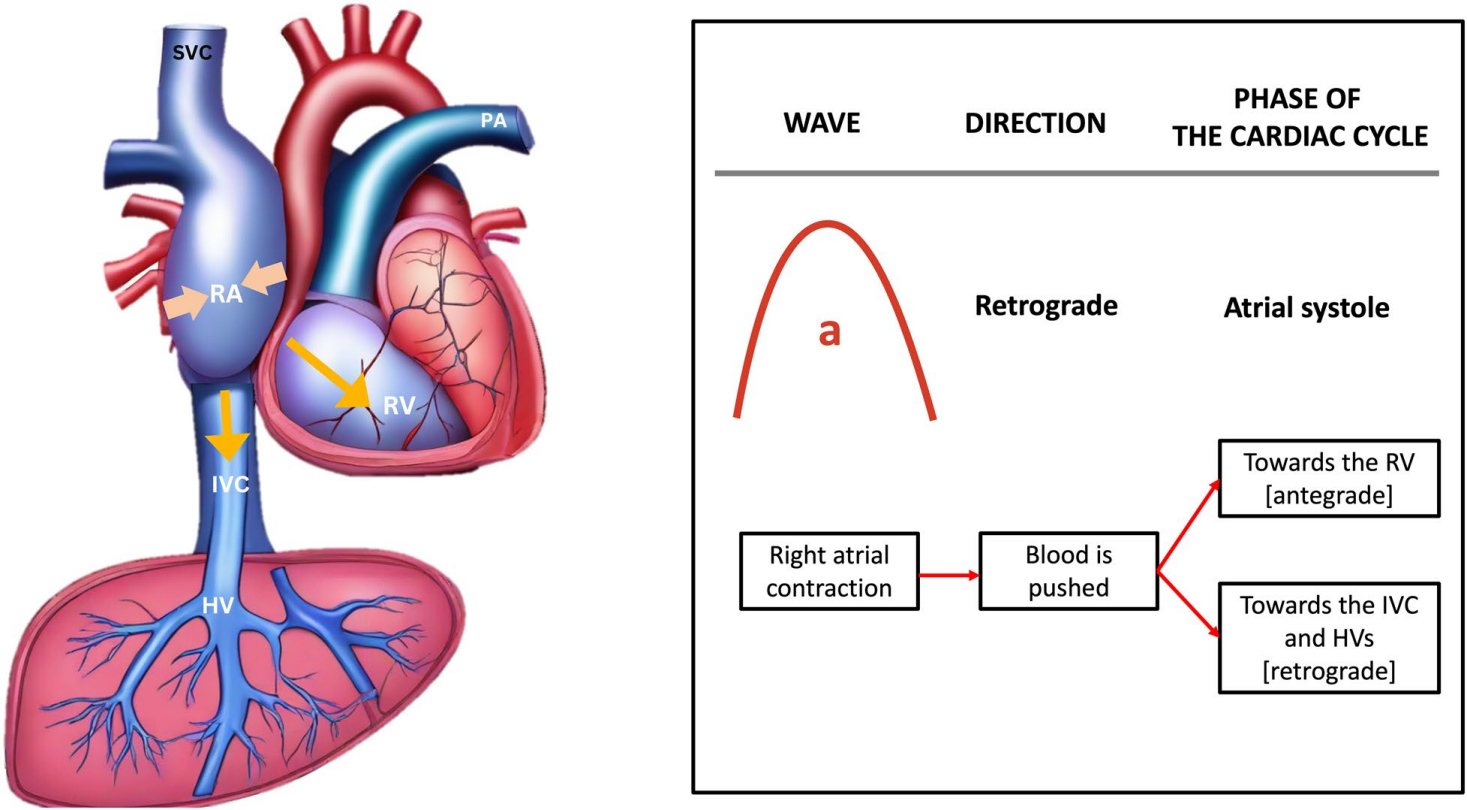
The A wave is generated by the contraction of the RA during atrial systole, increasing the RAP and pushing blood backward toward the liver. RA, right atrium; RAP, right atrial pressure; IVC, inferior vena cava; HV, hepatic vein; RV, right ventricle; SVC, superior vena cava; PA, pulmonary artery

The A wave is produced by the contraction of the right atrium during atrial systole, elevating the RAP and propelling blood backward (Fig. 13).

**Fig. 14:**
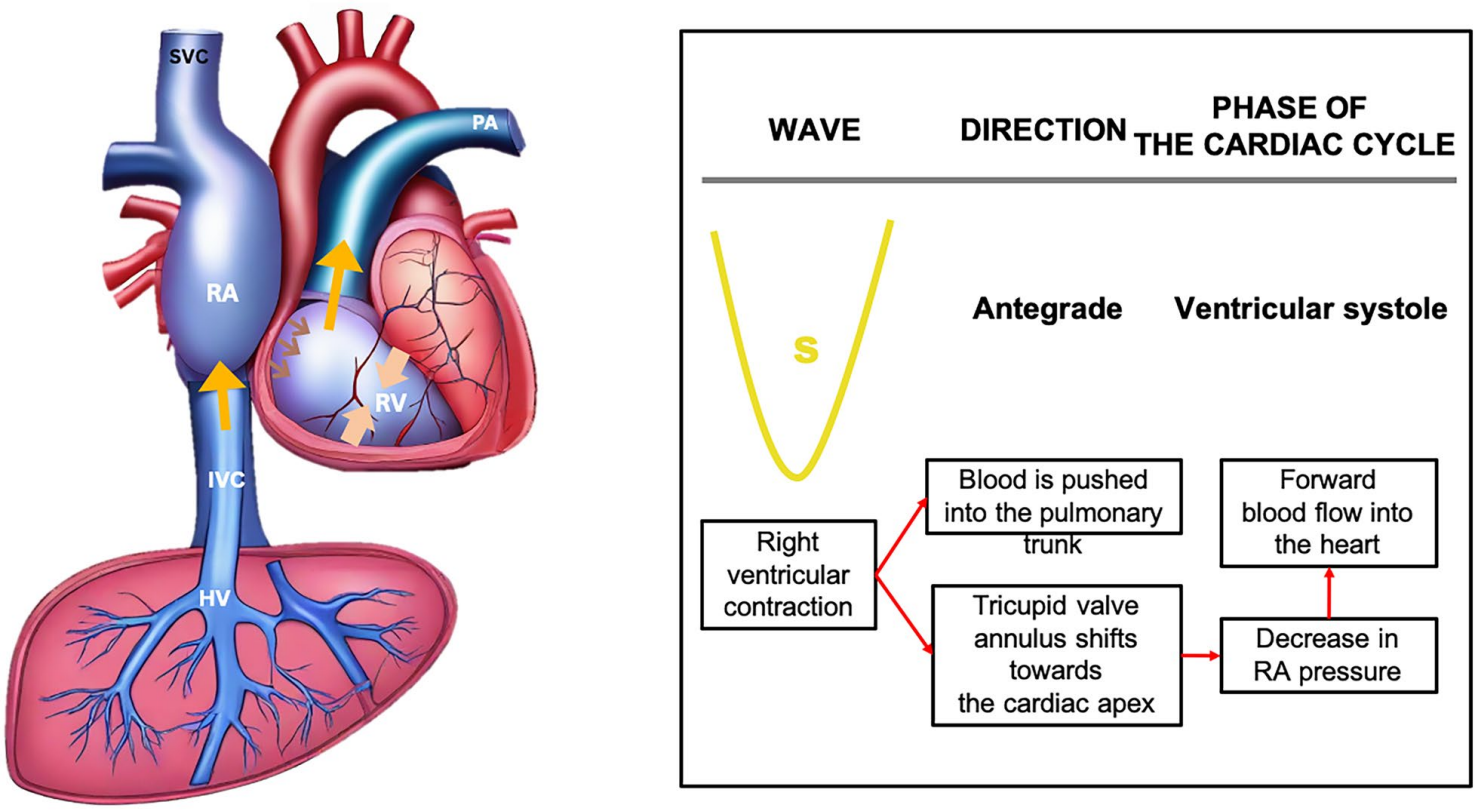
The S wave arises during ventricular systole when the tricuspid annulus moves towards the cardiac apex, directing blood flow from the HV into the IVC and RA, thereby generating the antegrade S wave. IVC, inferior vena cava; RA, right atrium; HV, hepatic vein; RV, right ventricle; SVC, superior vena cava; PA, pulmonary artery

The S wave occurs during ventricular systole, as the tricuspid annulus moves toward the ventricular apex, causing blood to flow from the HV into the IVC and right atrium, generating the antegrade S wave (Fig. 14).

**Fig. 15:**
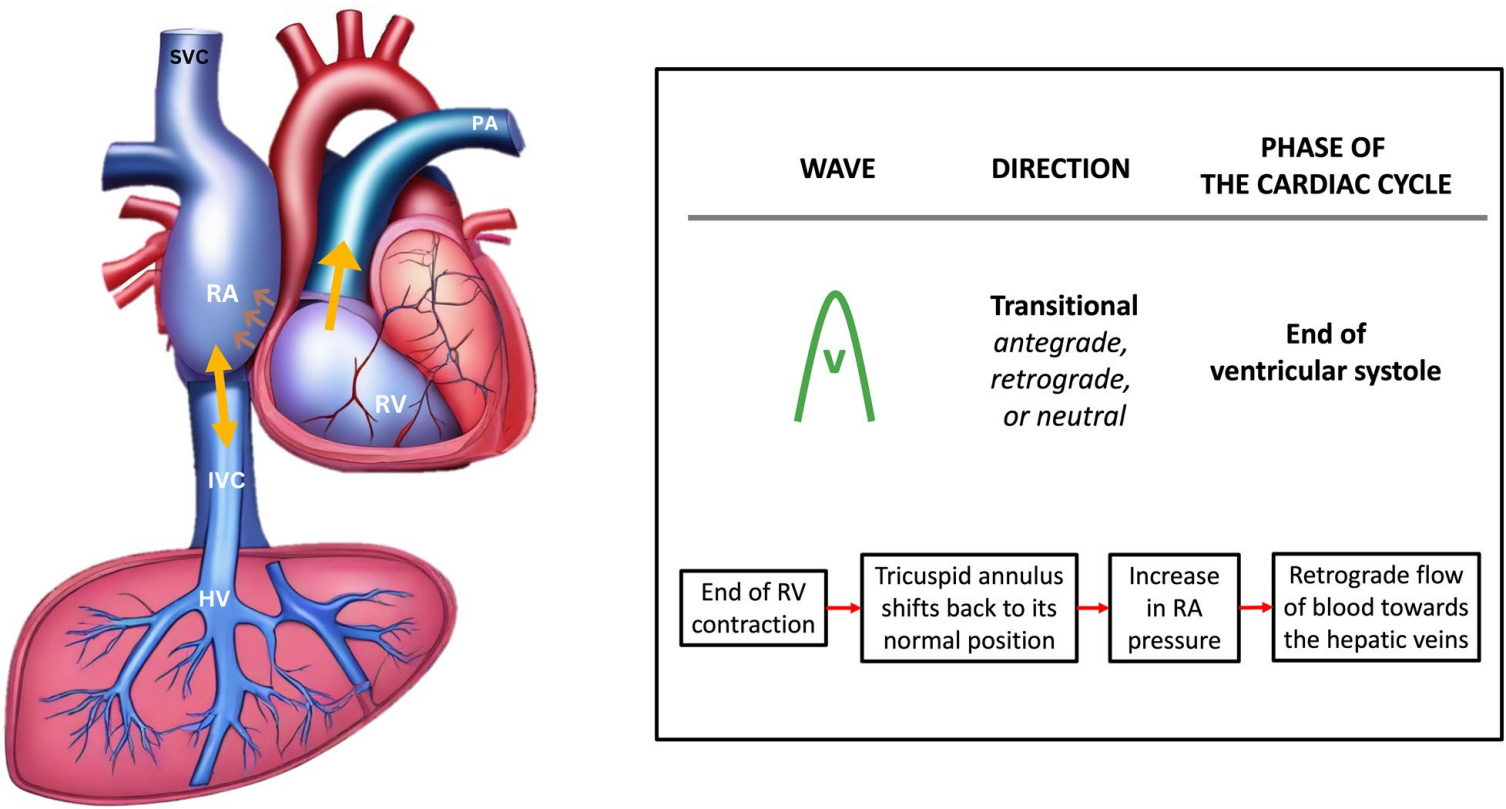
The V wave, appearing towards the end of ventricular systole, represents a transitional phase as the tricuspid annulus returns to its original position, inducing a retrograde wave that may manifest either above or below the baseline. RA, right atrium; IVC, inferior vena cava; HV, hepatic vein; RV, right ventricle; SVC, superior vena cava; PA, pulmonary artery

The V wave, occurring toward the end of ventricular systole, is a transitional wave resulting from the tricuspid annulus returning to its position, leading to a retrograde wave that can be either below or above the baseline (Fig. 15).

**Fig. 16:**
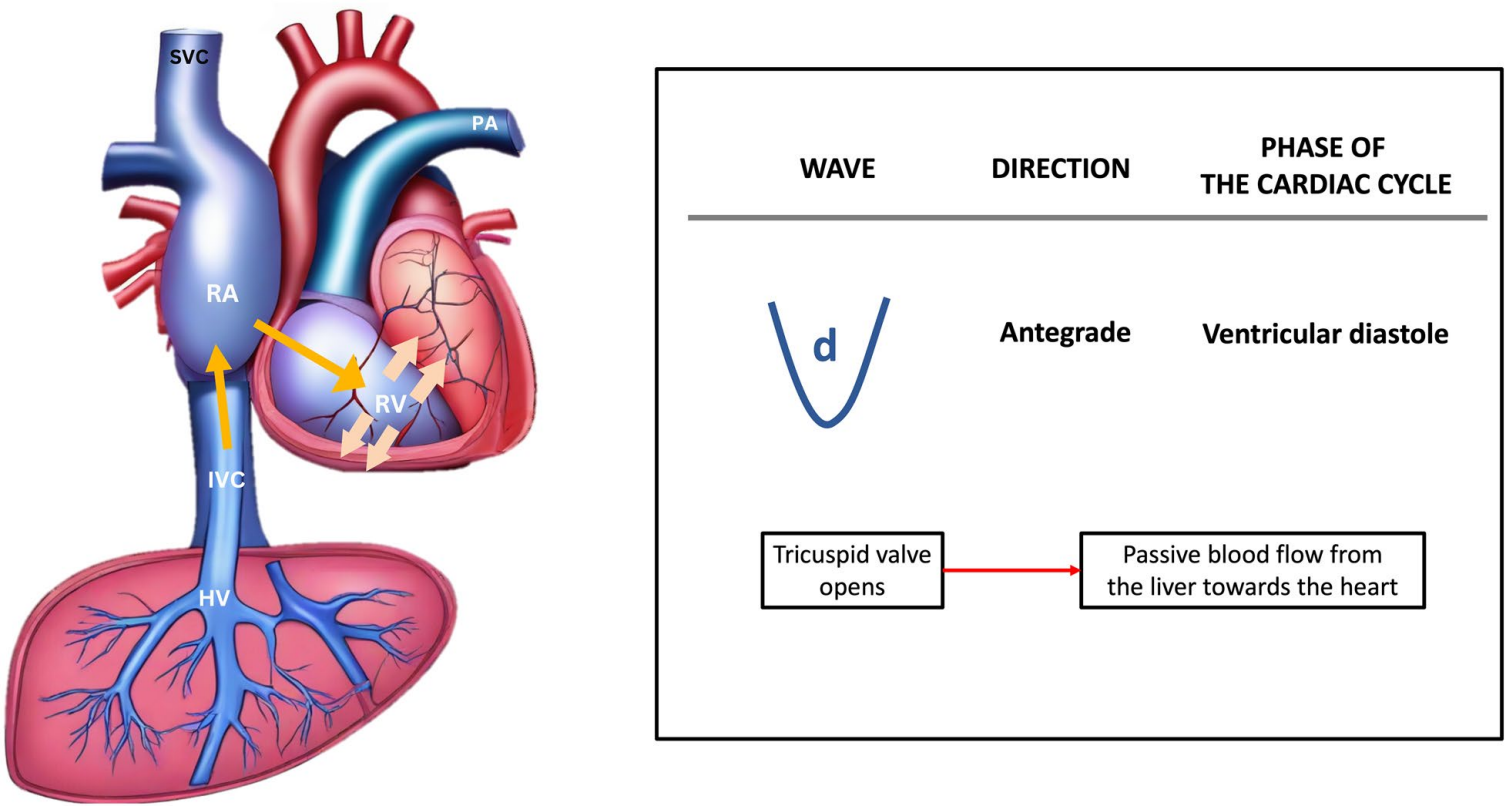
The D wave arises during ventricular diastole when the tricuspid valve opens, permitting blood to travel from the HV into the IVC and subsequently into the RA, thereby generating the antegrade D wave. HV, hepatic vein; IVC, inferior vena cava; RA, right atrium; RV, right ventricle; SVC, superior vena cava; PA, pulmonary artery

Finally, the D wave occurs during ventricular diastole, as the tricuspid valve opens, allowing blood to flow from the hepatic vein into the IVC and eventually entering the right atrium, thus generating the antegrade D wave (Fig. 16).

**Fig. 17:**
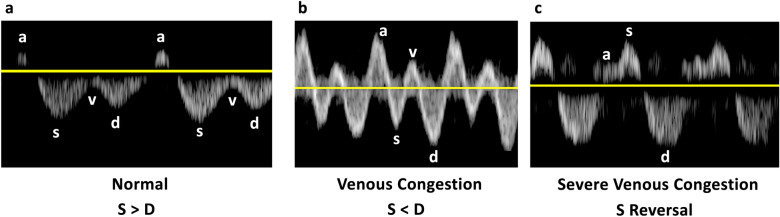
**a** Normally, the amplitude of the S-wave exceeds that of the D-wave. As the RAP increases in tandem with RV dysfunction and tricuspid regurgitation, **b** in mild to moderate venous congestion, the amplitude of the S-wave diminishes, becoming less than that of the D-wave, and **c** in severe venous congestion, the S-wave reverses its position, rising above the baseline, while only the D-wave remains below the baseline. RAP, right atrial pressure; RV, right ventricle

Normally, the amplitude of the S-wave is greater than that of D-wave. As the RAP increases alongside RV dysfunction and tricuspid regurgitation, the amplitude of the S-wave diminishes and may even reverse, positioning itself above the baseline, leaving only the D-wave below the baseline (Fig. 17) [4, 44].

### Potential pitfalls

**Fig. 18:**
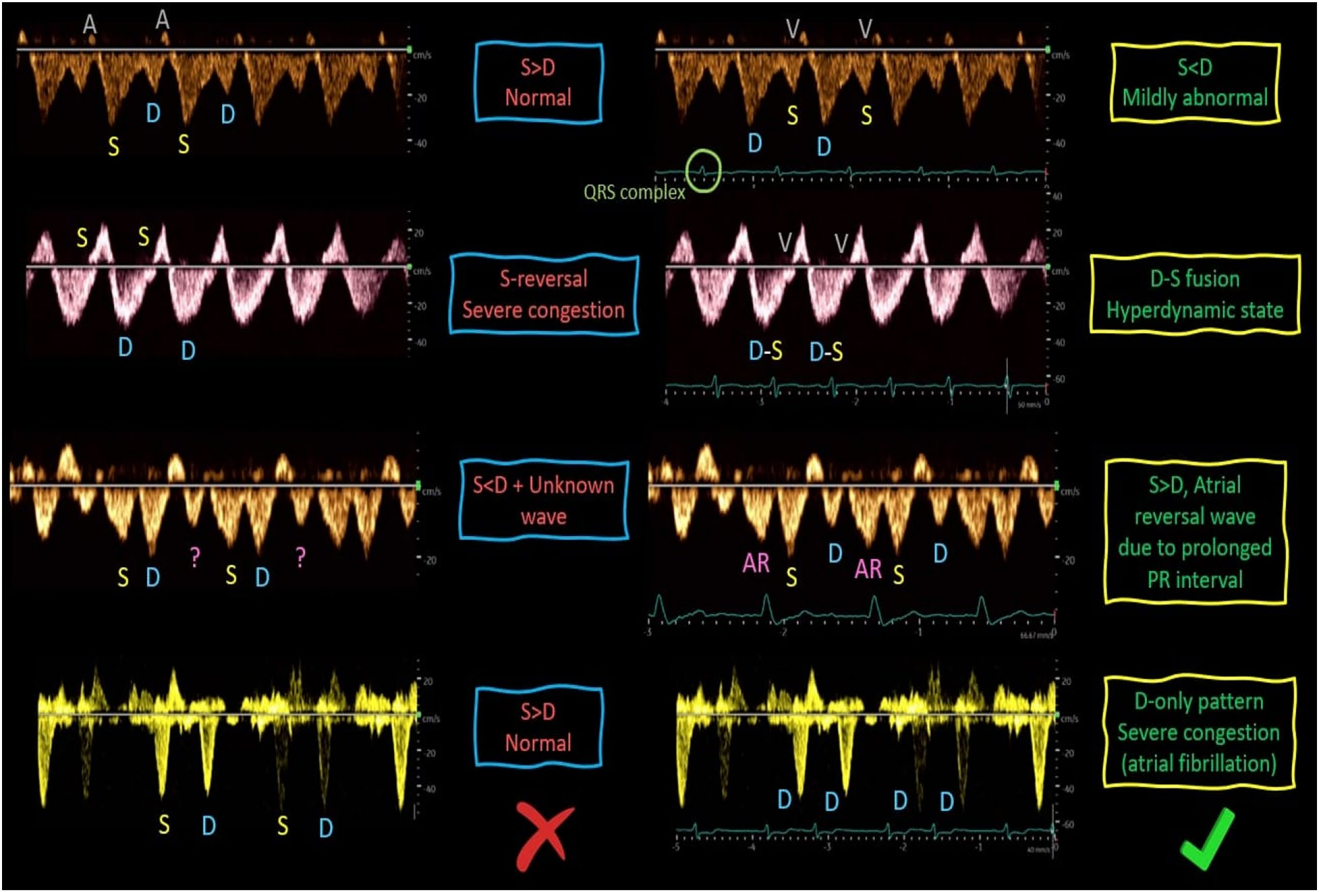
The HV waveform interpretation may be challenging without concurrent ECG tracing. HV, hepatic vein; ECG, electrocardiogram

**Fig. 19:**
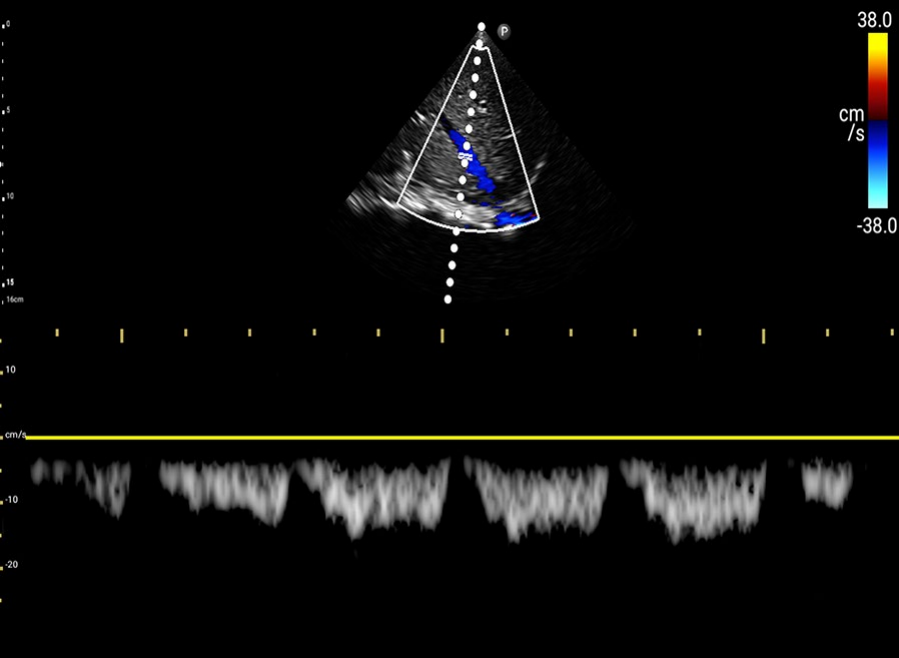
Blunting and reduction in cardiac phasicity may be observed in the HV waveform in conditions such as liver cirrhosis and fatty infiltration. HV, hepatic vein

- Atrial fibrillation can result in the absence of the A wave and a diminished S wave (S < D) without an increase in RAP [45]. Other rhythm abnormalities such as prolonged PR interval can lead to errors in interpretation without a simultaneous ECG (Fig. 18) [46].
- In conditions like liver cirrhosis and fatty infiltration, the hepatic vein waveform may exhibit blunting and a loss of cardiac phasicity (Fig. 19) [45].
- In cases of substantial structural tricuspid regurgitation, a persistent reversal of the S wave may occur, and this may not improve with volume reduction. It’s important to note that while this pattern does indicate venous congestion and may be associated with damage to congestive organs, it might not serve as an effective guide for deresuscitation.

## Portal vein Doppler

### Technique

**Fig. 20:**
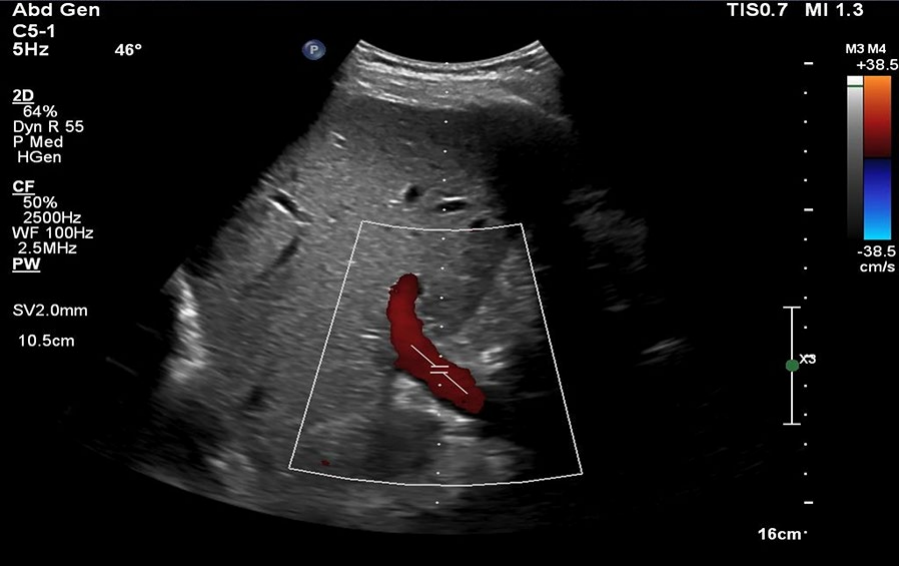
Pulsed wave Doppler mode with the sample volume (Doppler gate) positioned within the PV. PV, portal vein

**Fig. 21:**
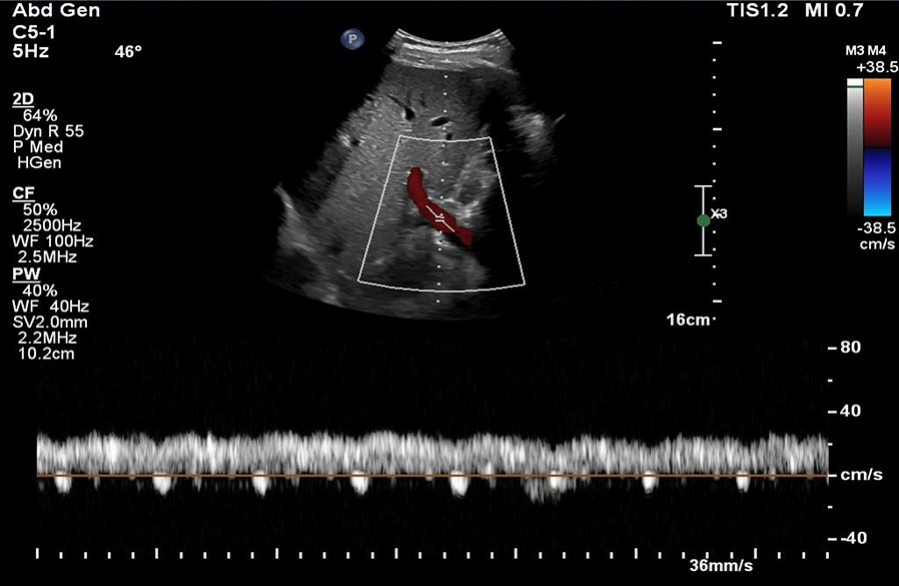
Pulsed-wave Doppler tracing of the PV. PV, portal vein

**Fig. 22:**
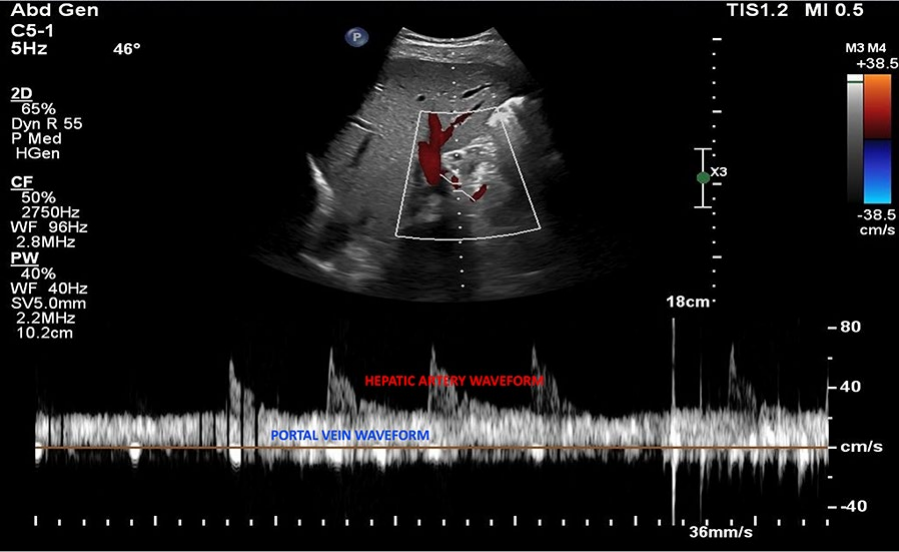
Pulsed-wave Doppler tracing of the PV with hepatic arterial flow interference. PV, portal vein

**Fig. 23:**
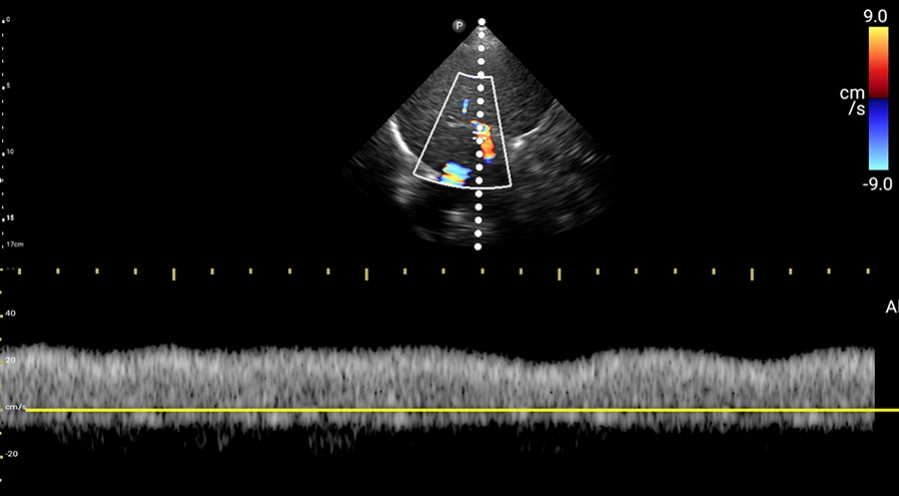
Pulsed-wave Doppler tracing of the PV without hepatic arterial flow interference. PV, portal vein

- Coronal view:
  - Like the HV Doppler, the coronal view offers a more distinct PV waveform, even though it can be accessed from the subxiphoid window.
  - Position the transducer in the same region as when capturing the hepatic vein waveform (Figs. 7, 8).
  - Slide it slightly in a caudal direction to observe the liver and adjacent right kidney (Video 11).
  - Fan the transducer anteriorly across the patient’s abdomen to unveil the portal vein (Videos 11, 12, 13).
- Doppler assessment:
  - If locating the portal vein proves difficult, employ color flow imaging to pinpoint its position (Videos 14, 15).
  - In a normal state, a continuous red flow is noted, signifying the movement of blood towards the probe (Video 14).
  - Engage the pulsed wave Doppler function and place the sample volume over the main portal vein for waveform visualization (Figs. 20, 21).
  - Decrease the Doppler scale to amplify the waveform amplitude, enhancing accuracy in calculating the portal vein pulsatility fraction (PVPF).
  - To prevent interference from the hepatic arterial flow (Fig. 22), position the sample volume distally within the portal vein (Fig. 23), avoiding the aliasing portion on color Doppler (aliasing refers to the mixture of colors indicating higher velocities, i.e., hepatic artery).

### Interpretation

**Fig. 24:**
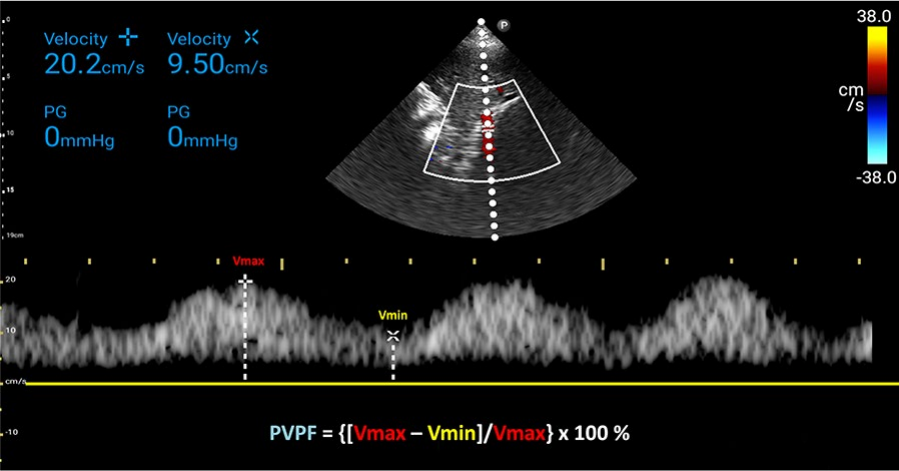
PVPF calculation. PVPF, portal vein pulsatility fraction; Vmax, maximum velocity; Vmin, minimum velocity

**Fig. 25:**
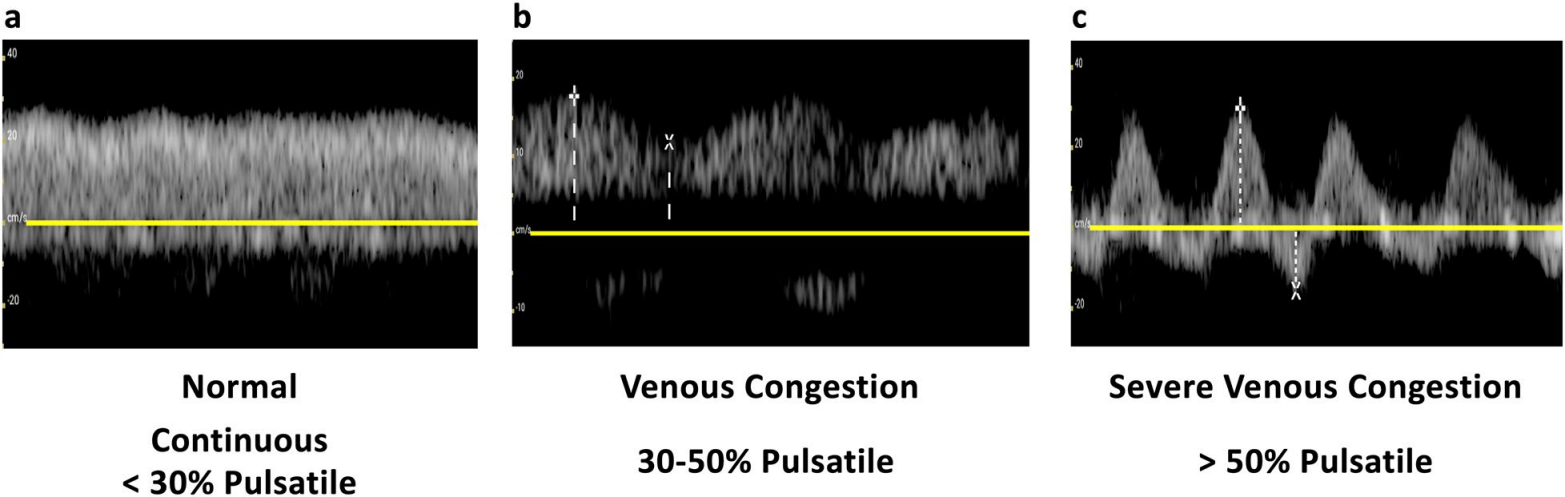
**a** The PV waveform typically exhibits a continuous pattern with limited pulsatility and a normal PVPF (< 30%) due to hepatic sinusoids that attenuate the linear transmission of RAP. **b** In mild to moderate venous congestion, characterized by elevated RAP and RV dysfunction, the PVPF may rise above 30% but remain below 50%. **c** A PVPF exceeding 50%, with or without systolic flow reversal (below the baseline), indicates severe congestion. PV, portal vein; PVPF, portal vein pulsatility fraction; RAP, right atrial pressure; RV, right ventricle

The PV waveform normally displays a continuous pattern with limited pulsatility, due to hepatic sinusoids that dampen the linear transmission of RAP [42, 47]. Normal portal vein pulsatility fraction (PVPF) ((Vmax − Vmin)/Vmax × 100%) is < 30% (Fig. 24). However, elevated RAP and RV dysfunction leading to venous congestion can increase the PVPF. A PVPF more than 30% but less than 50% suggests mild venous congestion, while a value surpassing 50%, with or without flow reversal (below the baseline) in systole, indicates severe congestion (Fig. 25) [20].

### Potential pitfalls

- Lean individuals and athletes may demonstrate an increased pulsatility index in the portal vein without a concurrent rise in RAP [48, 49].
- In patients with cirrhosis and portal hypertension, the PV waveform may display low-velocity continuous flow, or in severe cases, hepatofugal flow, i.e., complete flow reversal. Additionally, an increased PVPF may occur due to arteriovenous connections despite normal RAP [50, 51]. Conversely, in some of these patients, hepatic sinusoidal pressure transmission from the right atrium may be attenuated, potentially resulting in a normal PVPF despite elevated RAP and tricuspid regurgitation [52, 53]. Comparing with prior imaging, if available, can be helpful.
- Respiratory variations in the amplitude of the PV waveform might be mistaken for cardiac pulsatility, which, by definition, occurs in each cardiac cycle. Simultaneous ECG aids in differentiation.

## Intrarenal vein Doppler

### Technique

**Fig. 26:**
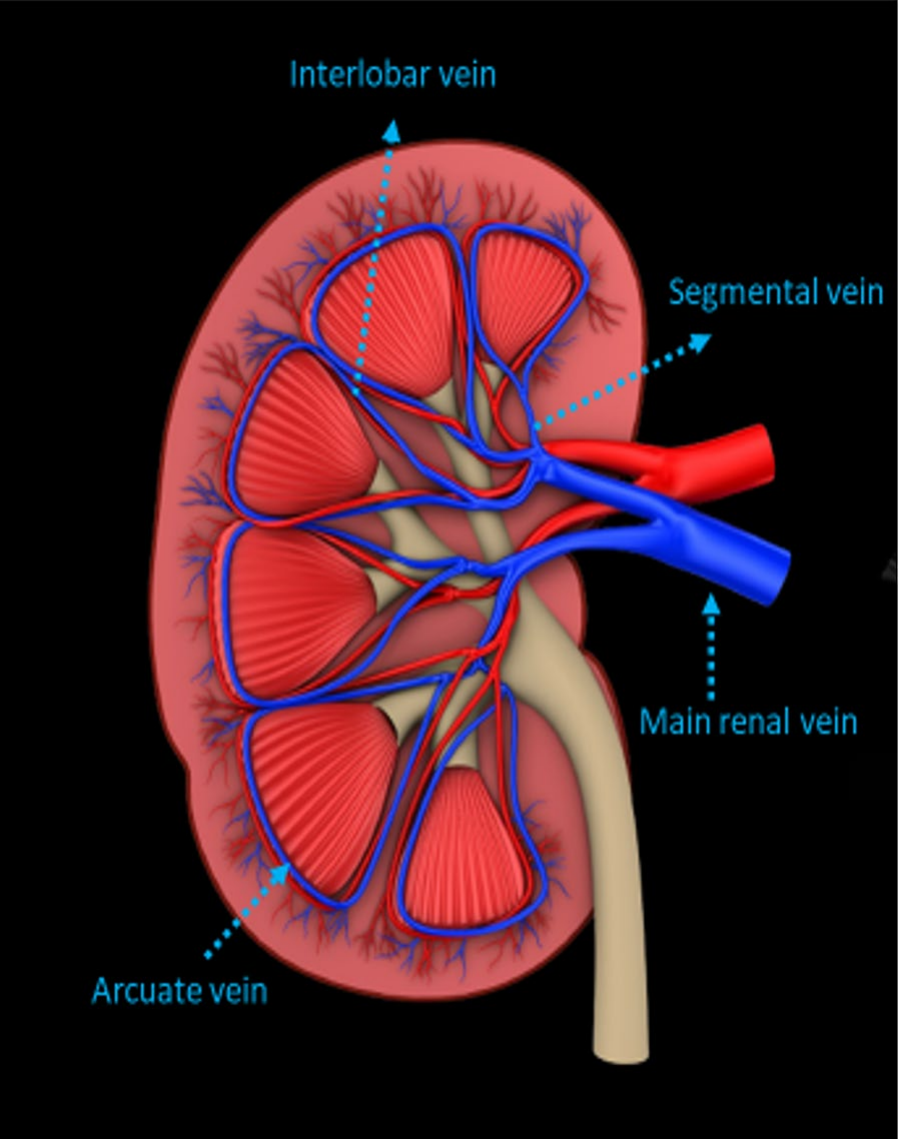
The major renal veins’ anatomy illustration

For VExUS assessment, the renal interlobar or arcuate veins are selected instead of the main renal vein or segmental vessels (Fig. 26) [20]. This is because veins located within the renal parenchyma provide insights into the effects of congestion and interstitial edema on renal perfusion. We prefer interlobar vessels due to their easier identification compared to arcuate veins and their alignment in parallel with the ultrasound beam.

**Fig. 27:**
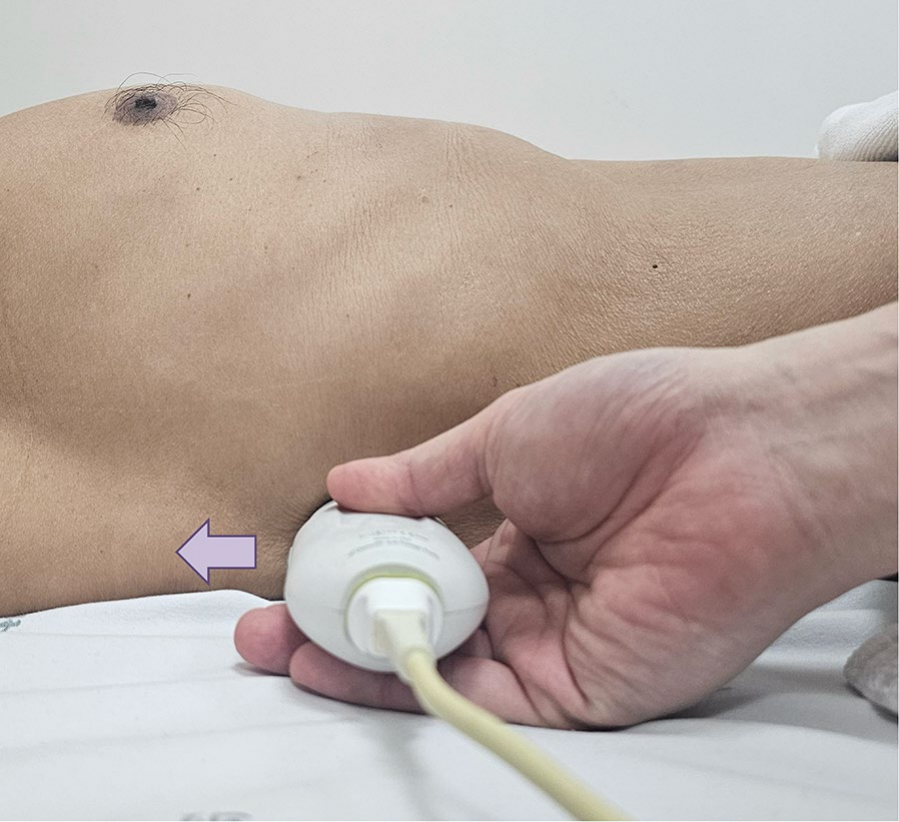
Position the transducer at the junction of an imaginary line extending from the xiphoid process to the postaxillary line, orienting the orientation marker towards the patient’s right axilla (arrow) to visualize the IRV in coronal view. IRV, intrarenal vein

**Fig. 28:**
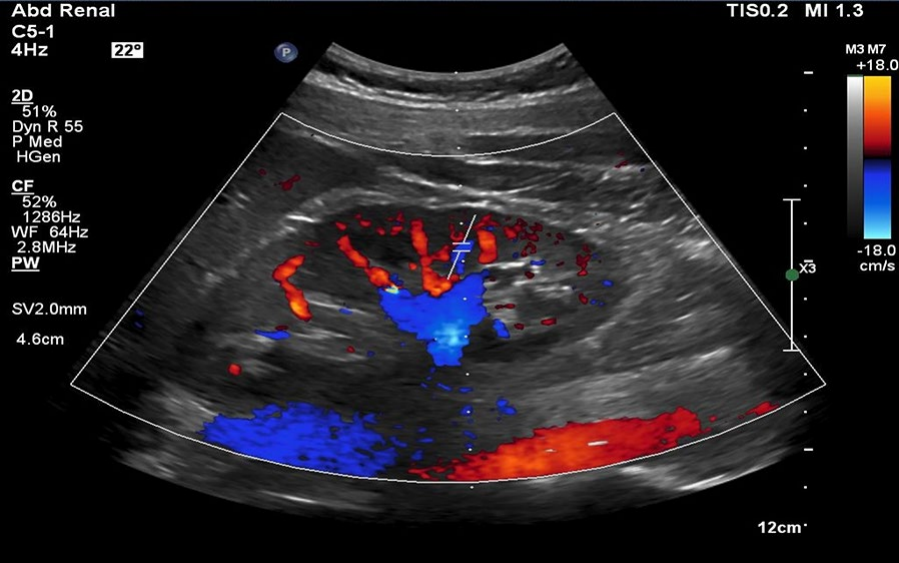
Pulsed wave Doppler mode with the sample volume (Doppler gate) positioned within the IRV. IRV, intrarenal vein

**Fig. 29:**
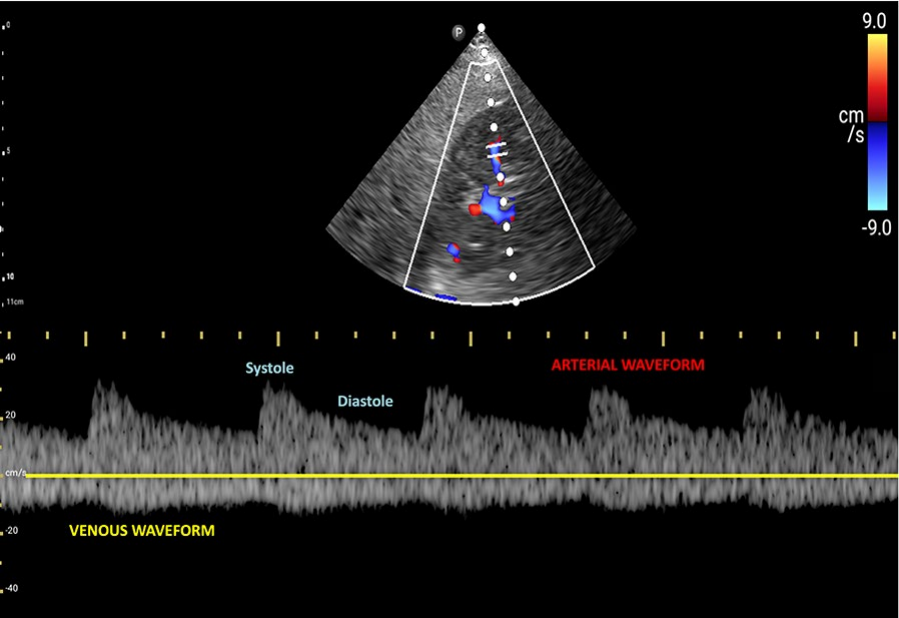
Pulsed-wave Doppler tracing of the IRV and the IRA. IRV, intrarenal vein; IRA, intrarenal artery

- Transducer position:
  Use the coronal window by placing the transducer at the junction of an imaginary line extending from the xiphoid process to the posterior axillary line, with the orientation marker directed toward the patient’s right axilla (Fig. 27).
- Pre-Doppler optimization:
  Slide the transducer slightly in a caudal direction to observe the kidney (Video 16), adjusting the depth to ensure proper visualization of the entire kidney. Next, utilize the zoom function to concentrate on the renal parenchyma, encompassing the cortex and medullary pyramids.
- Color Doppler:
  Engage the color Doppler and refine the box focus over the renal parenchyma to unveil the vessels (Video 17). The blood in the renal arterial system, flowing toward the transducer, appears red, while in the veins, flowing away from the transducer, it appears blue.
- Trouble shooting:
  If the vessels are not clearly visible:
  - Confirm abdomen preset is being used (as the cardiac preset may result in suboptimal flow).
  - Decrease the Doppler scale.
  - Gradually raise the color gain until the flow becomes visible, being careful to avoid speckling (Video 18).
  - Adjust the transducer tilt slightly upward or downward and observe if the color flow improves (Video 19).
  If no satisfactory improvement:
  - Consider using Power Doppler instead of color flow if available, as it is better at detecting low velocity flows.
  - Experiment with changing the angle of insonation (oblique or transverse views of the kidney are acceptable).
- Pulsed wave Doppler:
  Position the sample volume of the pulsed wave Doppler on the interlobar vessels to observe the intrarenal vein waveform, which is typically displayed below the baseline with the arterial waveform above (Figs. 28, 29).

### Interpretation

**Fig. 30:**
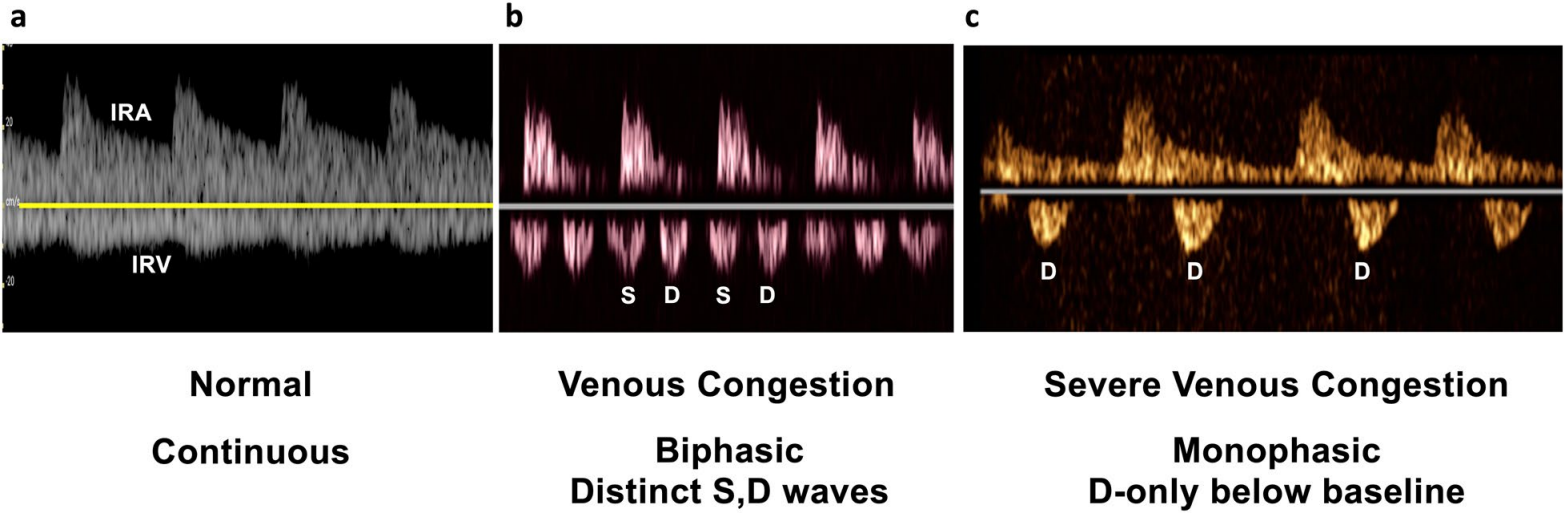
**a** Under normal conditions, the IRVD exhibits a continuous pattern with minimal pulsatility and no interruptions. **b** However, mild to moderate venous congestion results in heightened pulsatility and interruptions in the waveform, characterized by distinct systolic (S) and diastolic (D) waves. **c** In severe congestion, S-reversal occurs similarly to that of the HV, leaving only the D-wave below the baseline. IRVD, intrarenal vein Doppler; HV, hepatic vein; IRA, intrarenal artery; IRV, intrarenal vein

Under normal conditions, the IRVD exhibits a continuous pattern with minimal pulsatility and no interruptions (Fig. 29) [20]. However, venous congestion results in heightened pulsatility and interruptions in the waveform, marked by distinct systolic (S) and diastolic (D) waves, signifying mild to moderate congestion (Fig. 30) [20, 54]. The phases of the cardiac cycle are identified with the assistance of the simultaneous arterial waveform. In cases where the venous waveform is obtained in isolation, an ECG can help. In severe congestion, S-reversal occurs similar to that of HV leaving only D-wave below the baseline (Fig. 30) [20, 54].

### Potential pitfalls

- Technically difficult to acquire optimal images compared to HV and PV especially in patients who cannot hold their breath/follow instructions [55].
- Not studied chronic kidney disease or renal transplant recipients.

## VExUS grading system

**Fig. 31:**
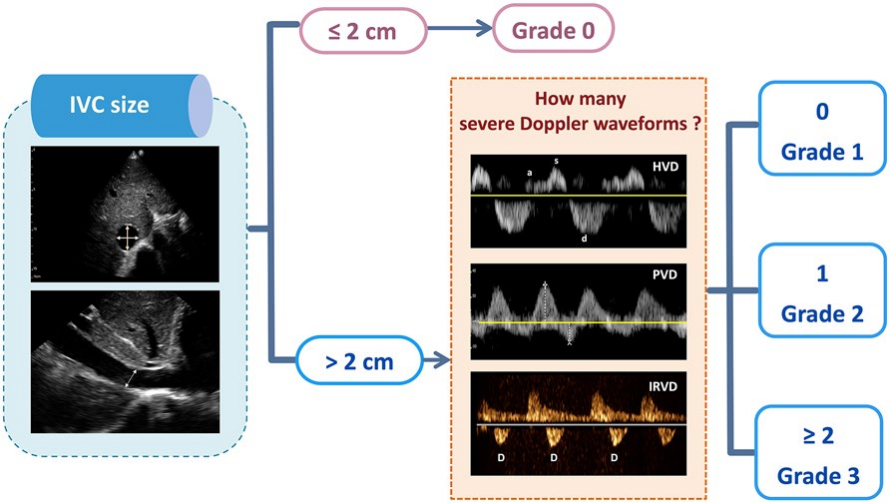
The VExUS grading system categorizes congestion based on IVC diameter and Doppler findings in HV, PV, and IRV. An IVC diameter ≤ 2 cm indicates grade 0 (no congestion). Grades 1–3 are defined by abnormalities in HV, PV, and IRV Doppler. Mild to moderate HVD abnormalities have S-wave < D-wave but still below baseline; severe abnormalities exhibit S-wave reversal. PVPF of 30–50% is mild to moderate, while > 50% is severe. IRVD is mild to moderate with pulsatility and distinct S/D waves, severe with monophasic D-only pattern. Grade 1 has no severe waveform, grade 2 has one severe waveform, and grade 3 has ≥ 2 severe waveforms, indicating severe congestion. VExUS, venous excess ultrasound; IVC, inferior vena cava; HV, hepatic vein; PV, portal vein; IRV, intrarenal vein; PVPF, portal vein pulsatility fraction; HVD, hepatic vein Doppler; PVD, portal vein Doppler; IRVD, intrarenal vein Doppler

The grading process in VExUS is succinctly illustrated here (Fig. 31). Briefly, an IVC diameter less than 2 cm corresponds to grade 0, indicating the absence of congestion. However, it is important to keep in mind the previously mentioned pitfalls regarding IVC size. It is acceptable to proceed with the rest of the VExUS exam if the IVC appears circular and plethoric suggestive of elevated RAP. When the IVC diameter exceeds 2 cm, three grades of congestion are defined based on the severity of abnormalities observed in hepatic, portal, and intrarenal venous Doppler. In HV Doppler, a mildly abnormal pattern is characterized by a systolic (S) wave smaller than the diastolic (D) wave but still below the baseline. It is deemed severely abnormal when the S-wave is reversed. Portal vein Doppler is considered mildly abnormal with a pulsatility ranging from 30 to 50% and severely abnormal when it is 50% or greater. IRVD is mildly abnormal when it is pulsatile with distinct S and D components and severely abnormal when it is monophasic with a D-only pattern. The absence of severely abnormal waveforms but presence of one or more mildly abnormal waveforms corresponds to a VExUS grade of 1. A single severe waveform aligns with grade 2. Finally, two or more severe waveforms indicate VExUS grade of 3 or severe venous congestion [20].

### Potential pitfalls and cautionary notes

- VExUS grading does not differentiate between venous congestion resulting from volume overload and pressure overload. It is essentially a bedside tool to assess the severity of organ congestion irrespective of the cause and monitor the response to decongestive therapy [22–27]. Management should consider the appropriate treatment for each patient, as volume removal may not always be the best course of action.
- VExUS should be interpreted within the clinical context and integrated with other bedside information, including clinical, laboratory, and imaging data. It should always be performed in conjunction with cardiopulmonary POCUS.
- For example, in patients with long-standing pulmonary hypertension and high VExUS score, caution is advised to avoid aggressive fluid removal, as their cardiac output may rely on high preload. Improvement in VExUS can sometimes be observed with the use of pulmonary vasodilators in the context of right ventricular dysfunction [28]. Similarly, excessive volume removal in a patient with high VExUS score, without recognizing that the congestion is due to pericardial effusion, may precipitate tamponade by reducing intracardiac pressure relative to pericardial compression.
- VExUS only indicates one component of the hemodynamic circuit and should not be used as a substitute for the detailed hemodynamic assessment.
- VExUS is not a tool to assess volume responsiveness.
- VExUS has been primarily investigated in cardiac surgery patients and individuals with decompensated heart failure. However, its effectiveness in different clinical subsets of patients is yet to be thoroughly assessed to ascertain its optimal integration into appropriate management strategies.

## Conclusion

VExUS is a valuable tool for noninvasive assessment of a patient’s hemodynamics at the bedside. Proficiency in acquiring optimal Doppler images and interpreting them in the appropriate clinical context is essential for POCUS users. Otherwise, incorrect patient management may ensue, posing the risk of potential harm to the patient.

## Supplementary Information

**Additional file 1. Video 1:** IVC short-axis view. Abbreviation: IVC, inferior vena cava.

**Additional file 2. Video 2:** Probe manipulation for transitioning the IVC from the short axis to the long axis. Abbreviation: IVC, inferior vena cava.

**Additional file 3. Video 3:** Transition from the IVC short axis to the IVC long axis. Abbreviation: IVC, inferior vena cava.

**Additional file 4. Video 4:** Probe manipulation to capture the long-axis view of the HV draining into the IVC. Abbreviations: IVC, inferior vena cava; HV, hepatic vein.

**Additional file 5. Video 5:** The long-axis view of the HV draining into the IVC. Abbreviations: IVC, inferior vena cava; HV, hepatic vein.

**Additional file 6. Video 6:** Slide the probe slightly towards the patient’s head to visualize the liver-diaphragm interface, then tilt the probe downward to visualize the HV. Abbreviation: HV, hepatic vein.

**Additional file 7. Video 7:** HV coronal view in cardiac preset. Abbreviation: HV, hepatic vein.

**Additional file 8. Video 8:** HV coronal view in abdominal preset. Abbreviation: HV, hepatic vein.

**Additional file 9. Video 9:** HV coronal view in cardiac preset with color Doppler imaging. Abbreviation: HV, hepatic vein.

**Additional file 10. Video 10:** HV coronal view in abdominal preset with color Doppler imaging. Abbreviation: HV, hepatic vein.

**Additional file 11. Video 11:** Slide the probe caudally to visualize the interface between the liver and right kidney, then tilt the probe upward to visualize the PV. Abbreviation: PV, portal vein.

**Additional file 12. Video 12:** PV coronal view in cardiac preset. Abbreviation: PV, portal vein.

**Additional file 13. Video 13:** PV coronal view in abdominal preset. Abbreviation: PV, portal vein.

**Additional file 14. Video 14:** PV coronal view in cardiac preset with color Doppler imaging. Abbreviation: PV, portal vein.

**Additional file 15. Video 15:** PV coronal view in abdominal preset with color Doppler imaging. Abbreviation: PV, portal vein.

**Additional file 16. Video 16:** Slide the transducer slightly caudally to visualize the kidney.

**Additional file 17. Video 17:** IRV coronal view in abdominal preset with color Doppler imaging. Abbreviation: IRV, intrarenal vein.

**Additional file 18. Video 18:** IRV with color Doppler imaging as color gain gradually increases. Abbreviation: IRV, intrarenal vein.

**Additional file 19. Video 19:** Transducer manipulation with tilt slightly upward or downward is performed to observe if the color flow improves.

## Data Availability

The data used and/or analyzed during the current study are available from the corresponding author on reasonable request.

## Abbreviations

RAP: Right atrial pressure
RV: Right ventricle
AKI: Acute kidney injury
POCUS: Point of care ultrasound
Pmsf: Mean systemic filling pressure
VExUS: Venous excess ultrasound
IVC: Inferior vena cava
ICU: Intensive care unit
ECG: Electrocardiogram
HV: Hepatic vein
PV: Portal vein
FAST: Focused assessment with sonography in trauma
IRVD: Intrarenal vein Doppler
PVPF: Portal vein pulsatility fraction
